# Genome-Wide Identification and Capsaicinoid Biosynthesis-Related Expression Analysis of the *R2R3-MYB* Gene Family in *Capsicum annuum* L.

**DOI:** 10.3389/fgene.2020.598183

**Published:** 2020-12-21

**Authors:** Jin Wang, Yi Liu, Bingqian Tang, Xiongze Dai, Lingling Xie, Feng Liu, Xuexiao Zou

**Affiliations:** ^1^College of Horticulture, Nanjing Agricultural University, Nanjing, China; ^2^Engineering Research Center for Horticultural Crop Germplasm Creation and New Variety Breeding, Ministry of Education, Changsha, China; ^3^Longping Branch, Graduate School of Hunan University, Changsha, China; ^4^College of Horticulture, Hunan Agricultural University, Changsha, China; ^5^Hunan Vegetable Research Institute, Changsha, China

**Keywords:** Capsicum, *CaR2R3-MYB* family, capsaicinoid biosynthesis, expression analysis, co-expression

## Abstract

Capsaicinoids are naturally specialized metabolites in pepper and are the main reason that Capsicum fruits have a pungent smell. During the synthesis of capsaicin, MYB transcription factors play key regulatory roles. In particular, *R2R3-MYB* subfamily genes are the most important members of the MYB family and are critical candidate factors in capsaicinoid biosynthesis. The 108 *R2R3-MYB* genes in pepper were identified in this study and all are shown to have two highly conserved MYB binding domains. Phylogenetic and structural analyses clustered *CaR2R3-MYB* genes into seven groups. Interspecies collinearity analysis found that the *R2R3-MYB* family contains 16 duplicated gene pairs and the highest gene density is on chromosome 00 and 03. The expression levels of *CaR2R3-MYB* differentially expressed genes (DEGs) and capsaicinoid-biosynthetic genes (CBGs) in fruit development stages were obtained *via* RNA-seq and quantitative polymerase chain reaction (qRT-PCR). Co-expression analyses reveal that highly expressed *CaR2R3-MYB* genes are co-expressed with CBGs during early stages of pericarp and placenta development processes. It is speculated that six candidate *CaR2R3-MYB* genes are involved in regulating the synthesis of capsaicin and dihydrocapsaicin. This study is the first systematic analysis of the *CaR2R3-MYB* gene family and provided references for studying their molecular functions. At the same time, these results also laid the foundation for further research on the capsaicin characteristics of *CaR2R3-MYB* genes in pepper.

## Introduction

Secondary metabolites are important compounds in plants to resist stress, deter herbivores, and prevent attack from some pathogens (Tewksbury et al., 2008). It is clear that plants have evolved their own special secondary metabolites on the basis of adaptations to the surrounding environment. Amongst these, the unique defensive chemical compounds produced by Capsicum, including capsaicin (CAP), dihydrocapsaicin (DhCAP), and several analogs, collectively known as capsaicinoids (CAPDs), are the most widely involved (Stewart et al., 2007). It is also known that CAPDs, unique flavoring substances in chili peppers, make peppers spicy but also influence the synthesis and accumulation of volatile aroma substances (Aza-González et al., 2011). In addition to self-protection, CPADs are also widely applied across industries including food, pharmaceuticals, and medical areas (Naves et al., 2019).

Thus, regarding CPAD biosynthetic pathways, CAP and DhCAP account for nearly 90% in pepper species, divided into phenylpropanoid and branched chain fatty acid pathways (Choi et al., 2006). One specific approach is to synthesize capsaicin by condensing vanillylamine molecules and to derive this compound from phenylalanine *via* branched chain fatty acids (between 9 and 11 carbon atoms), themselves synthesized from either valine or leucine (Arce-Rodriguez and Ochoa-Alejo, 2019). Indeed, as sequencing technology has developed, studies have revealed that Capsicum fruit biosynthesis is strongly influenced by genotype-environment interactions (Qin et al., 2014). Capsaicinoid-biosynthetic genes (CBGs) are expressed preferentially as typical response factors, specifically in the pericarp and placenta, during pepper fruit development processes (Liu et al., 2013). Studies have identified several structural CBGs [such as, *CoMT*, *C4H*, *AT3*, *KAS*, putative aminotransferase (*pAMT*), and *Acl*] involved in capsaicinoid biosynthesis (Von Wettsteinknowles et al., 2000); these accumulate in epidermal cell vesicles in placental tissue and start accumulating between 10 and 20 days post anthesis (DPA), increasing between 20 and 40 DPA (Arce-Rodriguez and Ochoa-Alejo, 2017). Orthologous genes in the pathways of other solanaceous plants (e.g., tomato and potato) are rarely expressed at this stage (Kim et al., 2014). Genetic studies have revealed that two leaky *pAMT* alleles (*pamtL1* and *pamtL2*) as well as a loss-of-function *pAMT* allele reduce capsaicinoid levels (Tanaka et al., 2019), while mutations in acyltransferase (*Pun1*) and *pAMT* lead to disruption of the capsaicinoid biosynthesis putative gene ketoacyl-ACP reductase (*CaKR1*) and a loss of pungency (Koeda et al., 2019). It is also clear that *Pun1* encodes an acyltransferase necessary to biosynthesize capsaicinoid (Stewart et al., 2005), and silenced *AT3* negatively influences the transcription of CBGs (Arce-Rodriguez and Ochoa-Alejo, 2015). The bulk of CBGs exhibit tissue- and stage-specific expressions accompanying the gradual accumulation of capsaicinoids. The transcription factors *Erf* and *Jerf* within the complex ERF family are expressed early in fruit development and participate in regulation of the pungency phenotype in chili (Keyhaninejad et al., 2014). These observations show that transcription factors also participate and play key regulatory roles in capsaicin pathway synthesis and metabolism.

Myeloblastosis (MYB) is one of the most important and the largest transcription factor gene families (Dubos et al., 2002). The MYB gene is divided into four subfamilies based on incomplete MYB domain repeats (R), each containing about 52 amino acid residues. This group includes the 4R-MYB, 3R-MYB, R2R3-MYB, and MYB-related subfamilies which each contains a single or partial MYB-related repeat, respectively (Jia et al., 2004). Specifically, R2R3-MYB is the dominant subfamily, occurring in the largest numbers in most plants (Rosinski and Atchley, 1998). Different MYB-type family members have been identified in many species, including in *Arabidopsis thaliana* (196 members) (Dubos et al., 2002), and watermelon (*Citrullus lanatus*) (162 members, of which 89 are *R2R3-MYB* type genes) (Wang et al., 2020). Similarly, 559 *R2R3-MYB*s have been identified in Solanaceae, including 119 complete sequences in tomato (*Lycopersicon esculentum* Mill.) (Gates et al., 2016). These genes have a wide range of functions and play pivotal regulatory roles in the synthesis of capsaicin. Methyl jasmonate induced *CaMYB108* is also involved in the regulation of capsaicin biosynthesis and stamen development (Sun et al., 2019), while the silencing of this gene significantly reduces the expression of CBGs and capsaicinoid content. These observations showed that *MYB* genes are widely involved in the regulation of capsaicinoid biosynthetic pathway structural genes (Arce-Rodriguez and Ochoa-Alejo, 2017). Natural variations *MYB31* and its elite allele *WRKY9* can served as transcription regulation direct targets for pepper pungency levels. These pathways have determined the evolution of extremely pungent peppers (Zhu et al., 2019).

Currently, it remains unclear whether, or not, the members of the *R2R3-MYB* family have more genes involved in the capsaicinoid biosynthesis process in pepper (*Capsicum annuum* L.) and its regulatory network. Thus, 108 *CaR2R3-MYB* genes were identified in this study in both CM334 pepper and “Zunla-1” pepper genomes. Expression profiles in the pericarp and placenta were determined during fruit development, and co-expression networks of *CaR2R3-MYB* genes and CBGs were associated with gene structures, phylogenetic relationships, interspecies synteny, and *cis*-element compositions. The outcomes of this analysis imply that *Capana01g000495*, *Capana02g000906*, *Capana02g003351*, *Capana07g001604*, *Capana08g000900*, and *Capana08g001690* are candidate *CaR2R3-MYB* genes involved in capsaicin biosynthesis.

## Materials and Methods

### The Identification of *R2R3-MYB* Transcription Factors in Pepper

A high-quality draft genome sequence of both hot pepper *C. annuum* cv. CM334 (Criollo de Morelos 334) (*C. annuum* Cultivars in Mexico) and a Chinese inbred derivative “Zunla-1” (*C. annuum* Cultivars in China) were used as reference genomes in this study. A HMM profile of Myb_DNA-binding domain (PF00249) was downloaded from the Pfam database (Elgebali et al., 2019), while HMMER 3.0 was applied to identify *MYB* family members with *E-*values ≤ 0.01 threshold (Finn et al., 2011). Protein domains of R2R3-MYBs were validated *via* SMART-Normal online software (Letunic and Bork, 2018). Protein modeling was predicted using the SWISS-MODEL online tool (Schwede et al., 2003). Theoretical the isoelectric points (PI) and molecular weights (Mw) values were computed using the ExPaSy online tool (Gasteiger et al., 2003), and subcellular localization values were predicted using the Softberry service platform-ProtComp 9.0 (Predict the sub-cellular localization for Plant proteins) online tool^1^.

### Gene Structure, Motifs, and Phylogenetic Analysis

The MEME v5.1.0 online tool (National Institutes of Health, Bethesda, MD, United States) was used to investigate conserved domains. Gene structures were analyzed using the Gene Structure Display Server (Hu et al., 2015). Full-length protein sequences of CaR2R3-MYB from *C. annuum* were aligned by ClustalW method, and used Gblocks^2^ online website to extract the gaps. Using unrooted neighbor-joining phylogenetic tree method of MEGA-X with the bootstrap test replicated 1,000 times (Kumar et al., 2018). The genome of Chinese inbred derivative “Zunla-1” acquired from pepper databases was used as the reference genome^3^.

### Chromosomal Location and Synteny Analysis

MCScanX was used to perform gene synteny and collinearity analysis, with match score of 50, gap score of -3, match size of 5, and *E*-value of 1e^–10^ parameters to analyze and calculate in-species duplicated genes (Wang et al., 2012). The Circos based Perl approach shows both gene chromosome positions and the synteny relationship of the pepper *R2R3-MYB* family (Krzywinski et al., 2009). KaKs_Calculator 1.2 were used to estimate the synonymous (Ks) and non-synonymous (Ka) substitution rates (Zhang et al., 2006).

### *Cis*-Elements Analysis in Promoter Regions

The Bedtools software was used to select the length of 2.0 kb upstream sequence for each gene CDS sequence from its promoter region (Quinlan and Hall, 2010), and to examine *cis*-regulatory elements of promoter sequences by PlantCARE-Search for CARE website^4^. Plots are presented using Tbtools (Chen et al., 2020).

### Materials and Transcriptome Data Analysis

A high-generation inbred Capsicum line 6421 was used for pepper development experiments. The Pericarp between 10 and 60 DAP (numbered G1–G11), the placenta and seed between 10 and 15 DAP (numbered ST1 and ST2), and the placenta between 20 and 60 DAP (numbered T3–T10) were taken from pepper fruit. The raw data for the transcriptome analysis used in this study were downloaded from Pepper Hub (Liu et al., 2017). The quality of sequencing data was controlled by Fastqc (Brown et al., 2017), and Trimmomatic-0.36 was used to filter the quality of the test data and remove low-quality sequences (Bolger et al., 2014). HISAT2 was used to compare two terminal sequencing reads to the reference genome of “Zunla-1” (Kim et al., 2014). The number of counts was calculated by using FeatureCounts (Yang et al., 2013). The R v3.6.1 language package DESeq2 was used to standardize counts data (Varet et al., 2016). FPKM (fragments per kilobase of transcript per million mapped reads) values were calculated to represent the gene expression.

### Co-expression Analysis Based on RNA-Seq Data

The weighted Gene Co-Expression Network Analysis (WGCNA) package was used in R v3.6.1 language^5^. RNA-seq data were used to perform WGCNA analysis. The weighted gene correlation network analysis (WGCNA) method was used to construct a co-expression network. WGCNA analyzes the gene expression patterns of multiple samples through gene expression data (Langfelder and Horvath, 2008). By calculating the adjacent order function formed by the gene network and the difference coefficients of different nodes, the TOM similarity algorithm calculates the co-expression correlation matrix to express the gene correlation in the network. The correlation network diagram is drawn by extracting the non-weight coefficients (weight) of related *CaR2R3-MYB* and *CBGs* in the matrix. Cytoscape v3.6.0 was used to reveal a co-expression plot (Shannon et al., 2003).

### Real Time Fluorescence Quantitative PCR (qRT-PCR)

Total RNA extraction was carried out using the TransZol kit (TransGen Biotech, Inc., Beijing, China). cDNA reverse transcription refers to use of the HiScript^®^IIQ RT SuperMix for qPCR (+gDNA wiper) vazyme kit (Vazyme, Piscataway, NJ, United States). quantitative polymerase chain reaction (qRT-PCR) was carried out in LightCycle ^∗^ 96 Real-Time PCR System (Roche, Basel, Switzerland) with 25 μL reaction system. Three biological repeats and three technical repeats were used to calculate the relative quantification according to the Ct values collected by the instrument. The formula is: 2^−△△*Ct*^ =  2^−[(*Target gene control Ct*−^^*Target gene sample Ct*)−(*Reference gene control Ct*−*Reference gene sample Ct*)]^. The actin gene *Capana04g001698* was used as reference gene which was selected from pepper. The primers of six *CaR2R3-MYB* DEGs and four CBGs were developed by GenScript Real-time PCR (TaqMan) Primer and Probes Design Tool^6^, which were listed in Supplementary Table 3.

## Results

### Genome-Wide Identification of CaR2R3-MYB Genes

On the basis of a Hidden Markov Model (HMM) MYB profile, there were 216 *CaMYB* genes in both CM334 and Zunla-1 genomic databases. Amongst *CaMYB* genes, 108 *R2R3* type, two *R3* type, one *R4* type, and 105 *MYB-related* types were further classified by searching both Pfam and SMART databases within the pepper genome. Further, comparing *CaR2R3-MYB*s between Zunla-1 and CM334, 32 homologous gene pairs exhibited different chromosomal annotation information. *R2R3* type genes were selected for further analysis and dubbed *CaR2R3-MYB*. Thus, Zunla-1 *CaR2R3-MYB*s were mainly used for the remaining analysis of this study.

**FIGURE 1:**
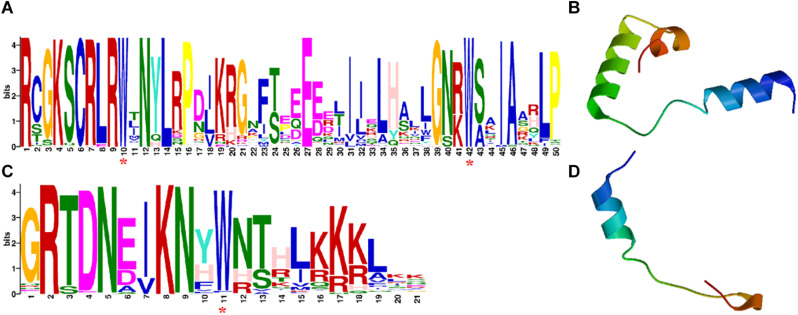
The domains of *CaR2R3-MYB* family genes and protein 3D structural models of R2 and R3 MYB repeats. **(A)** The R2 domain; **(C)** the R3 domain. The bit score indicates the information content for each position in the sequence while the purple asterisks below indicate the conserved tryptophan residues (Trp, W). **(B)** R2 repeats 3D structural model; **(D)** the R3 repeats 3D structure model.

All *CaR2R3-MYB*s contain two highly conserved MYB binding domains. The motif logo of *CaR2R3-MYB*s has 50 amino acid residues in the R2 repeat and 21 amino acid residues in the R3 repeat, respectively (Figures 1A,C). The HTH structure of these two domains as revealed by three-dimensional (3D) protein structural models showed that *CaR2R3-MYB*s matches the typical characteristics of the *R2R3-MYB* family (Figures 1B,D). Supplementary Table 1 showed that the PI of *CaR2R3-MYB*s range between 4.76 (*Capana06g000131*) and 10.18 (*Capana05g002248*), while the Mw range between 12.6148 KD (*Capana07g000392*) and 109.76643 KD (*Capana11g000012*). Subcellular localization prediction revealed that 101 *CaR2R3-MYB*s are located in the nuclear while three *CaR2R3-MYB*s are located in the cytoplasmic, three *CaR2R3-MYB*s are located in the mitochondrial region, and one *CaR2R3-MYB* (*Capana01g002201*) is located in the extracellular zone. There were 93.5% *CaR2R3-MYB*s are transcription factors, the functions of 5.6% *CaR2R3-MYB*s were related to the cytoplasm and mitochondrial organelles.

### Gene Structure, Motifs, and Phylogenetic Relationships of the *CaR2R3-MYB* Family

**FIGURE 2:**
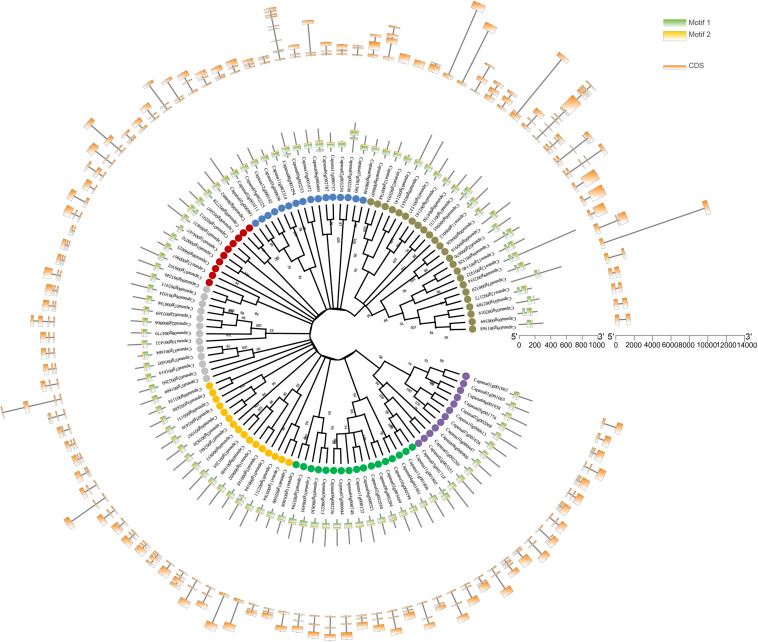
Phylogenetic relationships, conserved motifs, and gene structural analysis of *CaR2R3-MYBs*. Phylogenetic tree in the middle of 108 *CaR2R3-MYBs*. This is an unrooted phylogenetic tree constructed via the neighbor-joining (NJ) method with 1,000 bootstrap replicates. Colored dots distinguish seven groups, the distributions of conserved motifs in *CaR2R3-MYBs*. Two conserved putative motifs are indicated with green and yellow boxes. Exon/intron organization in *CaR2R3-MYBs* is in the outermost of the circle. Orange boxes represent coding sequence (CDS) regions while black lines show intron regions. Exon length can be inferred via the scale on the right side.

On the basis of statistically of high bootstrapping values, 108 *CaR2R3-MYB*s were separated into seven main groups in the unrooted phylogenetic tree based on protein sequences. All *CaR2R3-MYB* genes contained highly conserved MYB binding domains with two typical motifs, R1 and R2. Figure 2 showed the gene structure of exon-intron compositions on the outermost side of the circle. The numbers of exons range between 1 and 10 in *CaR2R3-MYB*s. Among them, 71 (65.7%) *CaR2R3-MYB*s have three exons, 22 (20.4%) *CaR2R3-MYB*s have two exons, seven *CaR2R3-MYB*s have four exons, four *CaR2R3-MYB*s have five exons, two *CaR2R3-MYB*s have five exons, and 10 exons and 11 exons have one *CaR2R3-MYB* each, respectively. These results revealed a high degree of sequence diversity which indicated that *CaR2R3-MYBs* may be related to formation mechanisms and evolutionary processes.

### Chromosomal Location and Interspecies Synteny Analysis

**FIGURE 3:**
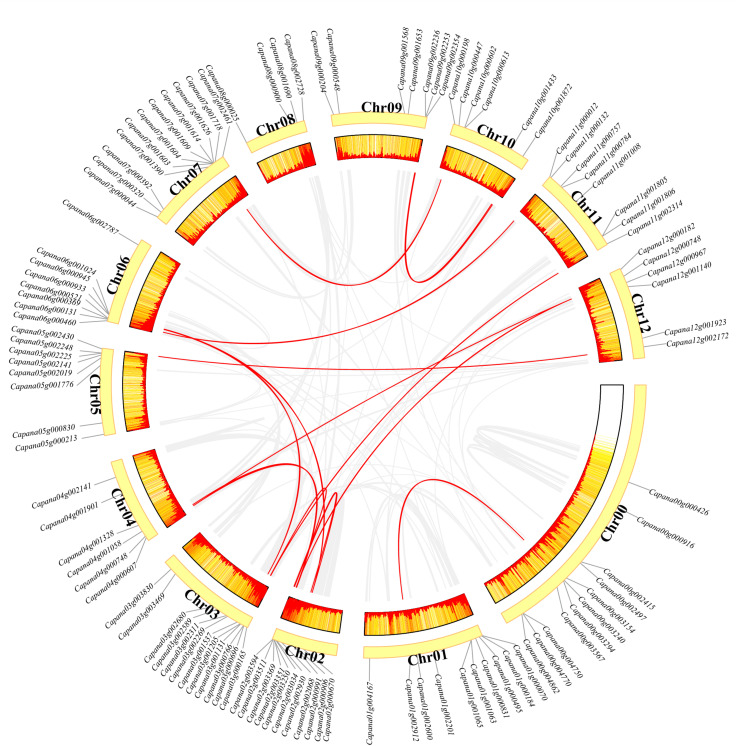
Chromosomal location, gene density, and interspecies synteny of *CaR2R3-MYBs*. The positions of *CaR2R3-MYB* genes in the pepper genome are marked on chromosomes. A heatmap shows the gene density of each chromosome. Red lines in the middle indicate duplication gene pairs of *CaR2R3-MYBs*, while grey lines indicate genome duplication gene pairs, and Chr refers to chromosome.

All *CaR2R3-MYB* genes were mapped onto the 12 different chromosomes of the pepper genome including the unclear information “00g” chromosome and “01g” to “12g” chromosomes. Chromosome 00 and chromosome 03 contained most *CaR2R3-MYBs* (12 genes), while chromosome 01 had 10 *CaR2R3-MYBs*, chromosome 02 and chromosome 07 harbored 11 *CaR2R3-MYBs*, chromosome 04, 10, and 12 contained six *CaR2R3-MYB* genes, chromosome 05, 06, and 11 had eight *CaR2R3-MYB*s, chromosome 08 harbored four *CaR2R3-MYBs*, and chromosome 09 contained seven *CaR2R3-MYBs* (Figure 3). A heatmap showed that gene density on chromosome 03 is the highest and no annotation genes are present at the front of chromosome 00.

The circos plot also revealed that 16 *CaR2R3-MYB* duplicated gene pairs are present. Non-synonymous mutation (Ka), synonymous mutation (Ks), and their ratios (Ka/Ks) were calculated to estimate selection pressure in duplicated genes. Ks values ranged between 0.46 and 2.3. In particular, *Capana03g000696-Capana11g002314* had no Ks value (NaN), indicating that duplication caused mutation at the nucleic acid level but that the amino acid sequence remained unchanged. The Ka/Ks values of the *CaR2R3-MYB* duplicated gene pairs ranged between 0.128 and 0.5 (Table 1). Indeed, all Ka/Ks values were less than 1.00 suggesting that *CaR2R3-MYB* duplicated genes have undergone purifying selection during the evolutionary process. Minimum Ks and maximum Ka/Ks values were observed between the duplicated gene pair *Capana03g001205-Capana06g000933*, indicating that these two genes might have experienced more purified selection.

**TABLE 1:** Duplication models for *CaR2R3-MYB* gene pairs in pepper.

Duplicate gene pair	Ka	Ks	Ka/Ks	AverageS-sites	AverageN-sites
*Capana00g003276-Capana01g002912*	0.191183	0.719625	0.26567	72.58333	245.4167
*Capana02g000906-Capana02g003369*	0.232616	0.816092	0.285037	189.6667	683.3333
*Capana02g000991-Capana02g003511*	0.124785	0.635704	0.196295	120.3333	464.6667
*Capana02g003369-Capana03g000766*	0.370969	2.304326	0.160988	193.75	688.25
*Capana02g003034-Capana04g000607*	0.275051	1.776355	0.15484	199.0833	610.9167
*Capana02g002930-Capana04g000748*	0.494677	1.560429	0.317013	196.6667	709.3333
*Capana02g000906-Capana06g001024*	0.390622	1.592982	0.245214	214.9167	772.0833
*Capana02g003034-Capana12g000748*	0.453205	1.669887	0.271399	161.6667	546.3333
*Capana03g001205-Capana06g000933*	0.236095	0.46796	0.50452	178.3333	700.6667
*Capana03g000696-Capana11g002314*	0.469324	NaN	NaN	164.25	654.75
*Capana04g000607-Capana12g000748*	0.251881	1.535706	0.164016	163.1667	562.8333
*Capana05g002444-Capana12g002172*	0.20509	1.593561	0.128699	124.1667	445.8333
*Capana06g000521-Capana11g000132*	0.359847	0.854725	0.421009	174.1667	689.8333
*Capana07g001626-Capana10g000447*	0.15329	0.858551	0.178545	199.6667	742.3333
*Capana09g002253-Capana10g001872*	0.143859	0.747546	0.192441	79.25	301.75
*Capana09g002354-Capana10g001956*	0.30921	1.488141	0.207783	220.4167	889.5833

*Ks, Ka, and Ka/Ks values are shown.*

### *CaR2R3-MYB* Putative *Cis*-Elements in Promoter Regions

**FIGURE 4:**
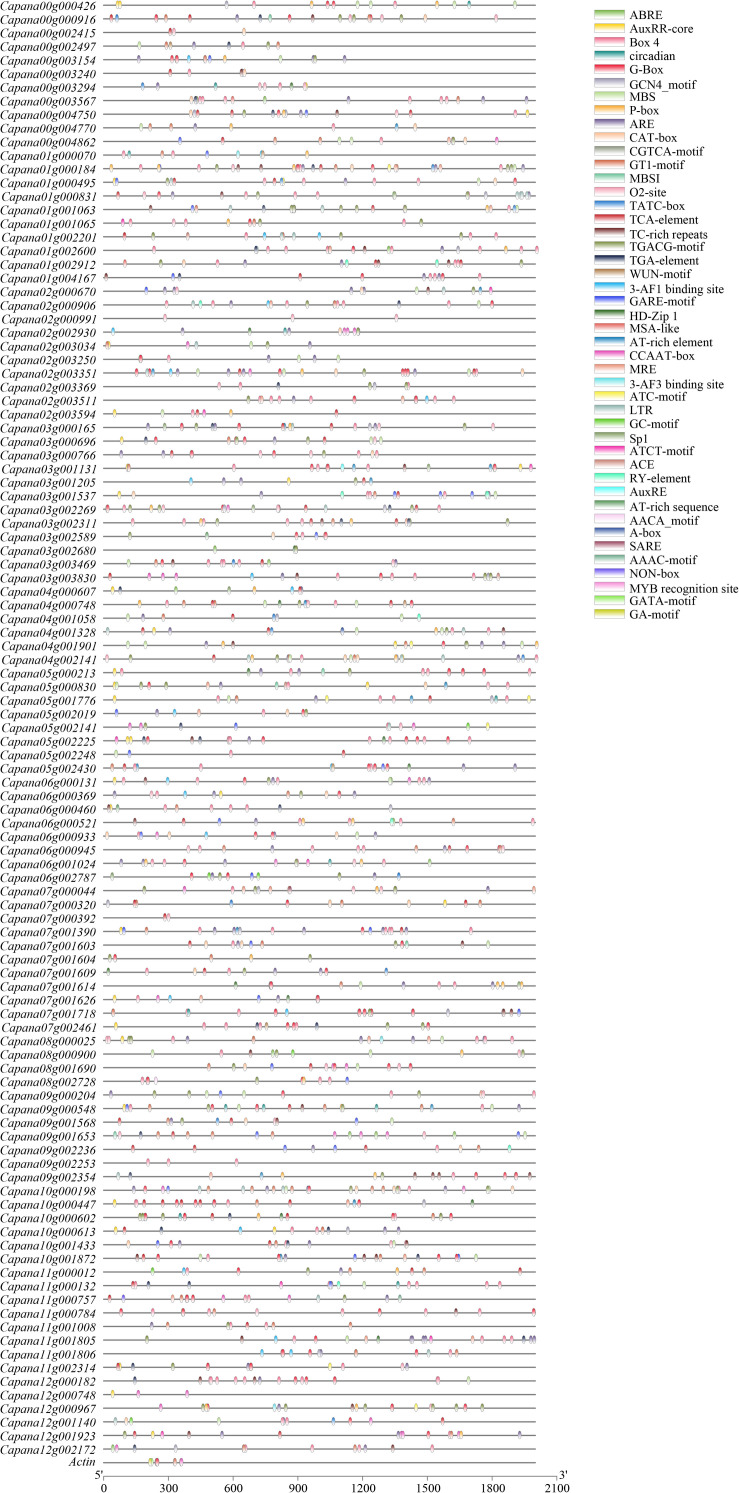
Predicted cis-elements in *CaR2R3-MYBs* promoters. Promoter sequences (−2,000 bp) of *CaR2R3-MYBs* and one actin gene were analyzed using PlantCARE. Different shapes and colors represent different elements. Annotations of cis-elements are listed in Supplementary Table S2.

The 2,000 base pairs (bp) upstream *CaR2R3-MYB* genes and actin gene sequences of the coding region were used to predict *cis*-regulatory elements *via* the PlantCARE online tool (Figure 4). A total of eight cellular development related *cis*-regulatory elements of *CaR2R3-MYB* genes were predicted on this basis, including meristem and endosperm expression, palisade mesophyll cells, flavonoid biosynthetic genes regulation, cell cycle regulation, and seed-specific regulation. There were 13 hormone-related *cis*-regulatory elements are also present, including abscisic acid, auxin, MeJA-, gibberellin-, and salicylic acid responsiveness as well as zein metabolism regulation. Similarly, 19 stress related *cis*-elements were also identified including light responsive elements, anaerobic induction, circadian control, anoxic specific inducibility, low-temperature responsiveness, defense and stress responsiveness, and wound-responsiveness. MBS and MRE are specifically MYB binding sites involved in drought-inducibility and light responsiveness (Supplementary Table 2). G-Box, ABRE, GT1-motif, MSA-like, and CCAAT-box were also present in the actin gene promoter region indicating that *cis*-elements are conserved in the promoter region of pepper genes.

### Expression of Capsaicinoid-Biosynthetic Genes and *CaR2R3-MYB* DEGs

**FIGURE 5:**
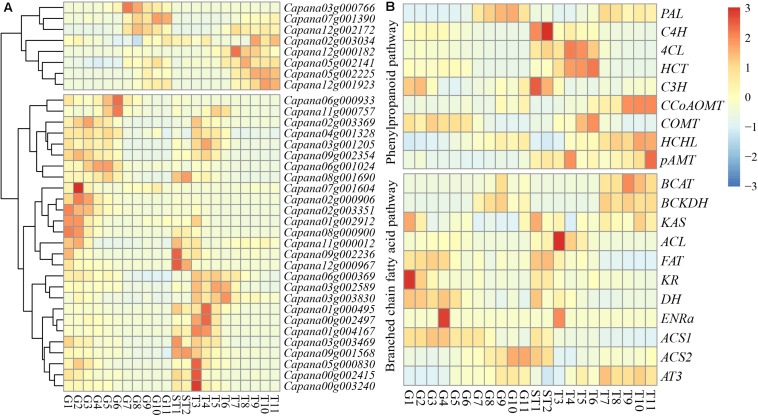
Expression patterns of *CaR2R3-MYB* DEGs and CBGs. Expression profiles using RNA-seq Fragments Per Kilobase Million (FPKM) data in the pericarp between ten DAP and 60 DAP (G1-G11), placenta and seed between ten DAP and 15 DAP (ST1 and ST2), and the placenta between 20 DAP and 60 DAP (T3-T10). **(A)** *CaR2R3-MYB* DEGs expression level **(B)** CBGs. CBGs including PAL: phenylalanine ammonia lyase; C4H: cinnamate 4-hydroxylase; 4CL: 4-coumaroyl-CoA ligase; HCT: hydroxycinnamoyltransferase; C3H: coumaroylshikimate/quinate 3-hydroxylase; CCoAOMT: caffeoyl-CoA 3-O-methyltransferase; COMT: caffeic acid O-methyl transferase; HCHL: hydroxycinnamoyl-CoA hydratase/lyase; pAMT: putative aminotransferase; in phenylpropanoid pathway, and BCAT: branched-chain amino acid transferase; KAS: ketoacyl-ACP synthase; ACL: acyl carrier protein; FAT: acyl-ACP thioesterase; ENR, enoyl-ACP reductase; KR, ketoacyl-ACP reductase; DH, hydroxyacyl-ACP dehydratase; ACS: acyl-CoA synthetase; AT: acyltransferase; in branched chain fatty acid pathway. The color scale at the left of each dendrogram represents log2 expression values, with red indicating high levels and blue indicating low levels of transcript abundance.

RNA-seq data from the pericarp between 10 days after germination (DAP) and 60 DAP, as well as from the placenta and seed between 10 and 15 DAP, and the placenta between 20 and 60 DAP were used to determine expression levels of *CaR2R3-MYB* DEGs and CBGs. A total of 35 (32.4%) *CaR2R3-MYB* DEGs of *CaR2R3-MYB* family genes from the pericarp between 10 and 60 DAP (adjust *P-*value < 0.01, | Log2foldchange| > 1) were identified. Nine DEGs were down-regulated while 26 genes were up-regulated (Supplementary Figure 1). The expression of *CaR2R3-MYB* DEGs can be separated into two groups (Figure 5A); the first part of expression levels was higher in late stage pericarp and placenta. There were eight *CaR2R3-MYB* DEGs in this part, of which seven were down-regulated genes. The expression of the other part was higher in the early stage of the pericarp, placenta and seed, and placenta, which contained 27 *CaR2R3-MYB* DEGs. Indeed, data suggested that *CaR2R3-MYB* genes are widely involved in the regulation of pepper fruit development process. The expression levels of CBGs in the capsaicinoid biosynthetic pathway were also identified (Figure 5B); *C3H*, *COMT*, *KAS*, *FAT*, *KR*, *DH*, *ENRa*, and *ACS1* were highly expressed in the early stage of the pericarp. Throughout the placenta and seed development process, *C4H*, *C3H*, *FAT*, and *DH* were significantly expressed between 10 and 15 DAP. Data showed that *4CL*, *HCT*, *ACL*, and *ENRa* were highly expressed in the early stage of the placenta, while *CCoAOMT*, *HCHL*, *pAMT*, *BCAT*, *BCKDH*, *KAS*, and *AT3* were highly expressed in the late stage of the placenta.

### Co-expression Analysis of Capsaicinoid-Biosynthetic Genes and *CaR2R3-MYBs*

**FIGURE 6:**
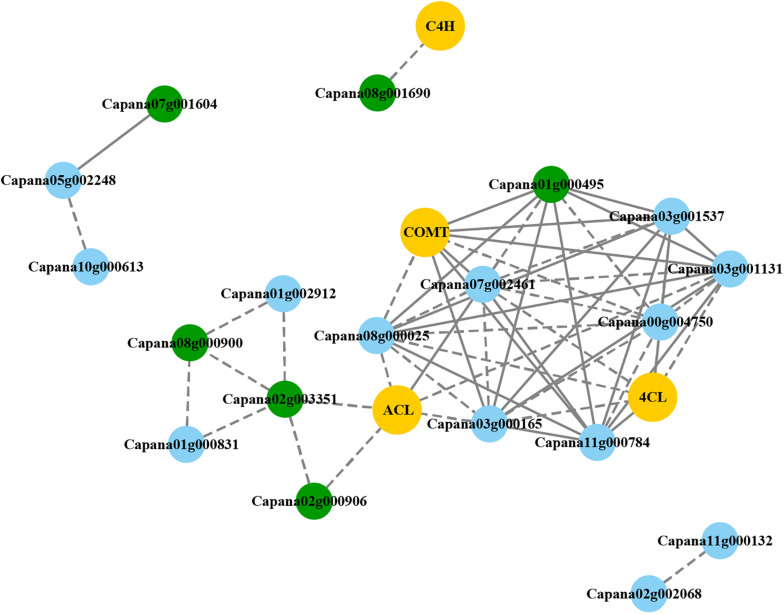
Co-expression networks of *CaR2R3-MYB* DEGs and CBGs. Yellow boxes denote CBGs, blue boxes denote *CaR2R3-MYB* genes, and green boxes denote *CaR2R3-MYB* DEGs. The solid line indicates that the co-expression weight is higher, while the dotted line indicates that the co-expression weight is lower.

The expression levels of 108 *CaR2R3-MYB*, 35 *CaR2R3-MYB* DEGs, and 20 CBGs were used to predict candidate *CaR2R3-MYB* genes related to capsaicin synthesis. As shown in Figure 6, **19** *CaR2R3-MYB* genes are co-expressed with four CBGs. Indeed, in the phenylpropanoid pathway, *C4H* was co-expressed with *Capana08g001690*, while *COMT* was highly co-expressed with *Capana07g002461*, *Capana03g000165*, *Capana11g000784*, *Capana03g001537*, and *Capana00g004750.* Similarly, *4CL* was co-expressed with *Capana11g000784*, and *Capana03g001537*. In a branched chain fatty acid pathway, *ACL* was co-expressed with *Capana02g003351*, *Capana02g000906*, *Capana07g002461*, *Capana03g000165*, *Capana11g000784*, *Capana03g001537*, *Capana01g000495*, *Capana08g000025*, *Capana03g001131*, and *Capana00g004750*. Six *CaR2R3-MYB* genes (*Capana01g000495, Capana02g000906, Capana02g003351, Capana07g001604, Capana08g000900*, and *Capana08g001690*) in the co-expression network were DEGs in the pericarp between 10 and 60 DAP. Data showed that these six *CaR2R3-MYB* DEGs are the candidate genes involved in capsaicin or capsanthin synthesis processes.

**FIGURE 7:**
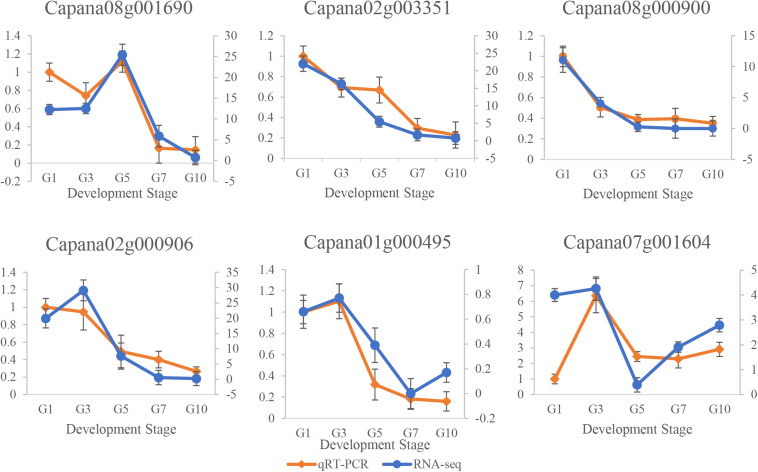
qRT-PCR and RNA-seq data of six candidate *CaR2R3-MYB* genes. Pericarp samples from G1, G3, G5, G7, and G10 show a series of expression changes in candidate *CaR2R3-MYB* genes. The orange line represents the qRT-PCR level while the blue line represents the RNA-seq level. CBGs are shown in Supplementary Figure S2.

The real-time qRT-PCR was performed to analyze the transcription levels of *Capana01g000495, Capana02g000906, Capana02g003351, Capana07g001604, Capana08g000900*, and *Capana08g001690* (Figure 7). Results illustrated that qRT-PCR and RNA-seq expression levels were similar for all six *CaR2R3-MYB* DEGs (Supplementary Figure 2). In terms of progressing fruit development, expression levels of *Capana02g003351* and *Capana08g000900* kept decreasing, while expression levels of *Capana01g000495, Capana07g001604, Capana02g000906*, and *Capana08g001690* first increased and then decreased. It is revealed that these six *CaR2R3-MYB* genes play key roles as candidate genes in capsaicin synthesis.

## Discussion

### Identification and Characterization of *CaR2R3-MYB* Genes

Pepper (*Capsicum*) is famous for its spiciness and is an economically important Solanaceae family crop cultivated globally for its nutritional benefits. Reference genome sequencing of the two varieties of pepper cultivars, CM334 (Mexico), and Zunla-1 (Guizhou, China), was completed in 2014 (Kim et al., 2014; Qin et al., 2014). In the pepper genome, however, gene families have been widely identified *via* incomplete annotation files. A total of 104 *CaNAC* genes (Diao et al., 2018), 35 *mTERF* genes (*CamTERF*s) (Tang et al., 2019), nine *CaCBL*, and 26 *CaCIPK* genes in the pepper genome (Ma et al., 2019). Here, 108 *CaR2R3-MYB*s were identified in both the CM334 and Zunla-1 genome, less than those identified in *Arabidopsis thaliana* (Stracke et al., 2001). The Basic Local Alignment Search Tool Protein (BLASTP) was used to align homologous genes in these two genomes. Thus, 11 *CaR2R3-MYB*s had “00g” chromosomal records in the Zunla-1 genome (also annotated in the CM334 genome), while seven *CaR2R3-MYB*s had chromosomal annotation in the Zunla-1 genome but no records in the CM334 genome (Supplementary Table 1). The complementarity of CM334 and “Zunla-1” can contribute to improve the complete annotation of the pepper genome. A chromosomal location circos plot illustrates that *CaR2R3-MYB*s are distributed evenly among every chromosome.

In order to study the evolution and transcriptional features of *CaR2R3-MYB*s, the number and distribution of introns and exons were analyzed. The number of CDS in the *CaR2R3-MYB* gene varies between 1 and 10, with the largest number of genes in three exons. Genes within the same subclass have similar structures and predicted motifs. These results indicate that the gene structure of *CaR2R3-MYB*s is highly conserved; a total of 16 *CaR2R3-MYB* duplicated gene pairs were identified *via* interspecies synteny analysis. The existence of duplicated genes is the main reason for *CaR2R3-MYB* gene amplification, one reason for the large number of family genes (Wang et al., 2013). A phylogenetic tree was constructed using cluster analysis such that *CaR2R3-MYB*s were divided into seven subclasses. As a result of highly conserved features, *CaR2R3-MYB*s within the same subclass tended to have similar functions.

Different *cis*-regulatory elements in the promoter sequences of genes may produce different expression patterns (Islam et al., 2019). A total of eight cellular development related *cis*-regulatory elements, 19 stress related *cis*-elements, and 13 hormone-related *cis*-regulatory elements were present. This analysis demonstrates that most *CaR2R3-MYB*s have divergent regulatory elements compared with the actin gene. The *CaR2R3-MYB* gene family has highly different *cis*-regulatory elements in the promoter region, which may lead to *CaR2R3-MYB* gene functional divergence at the transcriptional level (Haberer et al., 2004).

### Capsaicin Biosynthesis Related Expression Level of *CaR2R3-MYBs*

*R2R3-MYB* is recognized as the dominant MYB type gene with the largest number of members in most plants, widely involved in the regulation of plant morphogenesis, growth, metabolism, developmental processes, and responses to biotic or abiotic stresses (Rosinski and Atchley, 1998). In pepper plants, *R2R3-MYB* transcription factors also play significant roles. Virus-induced gene silencing has revealed that *MYB* and *WD40* are involved in the regulation of anthocyanin biosynthesis in chili pepper fruits (Aguilar-Barragan and Ochoa-Alejo, 2014), while the *R2R3-MYB* transcription factor and homolog gene may play a major role in abiotic stress signaling pathways (Seong et al., 2008). Three *R2R3-MYB* transcription factor genes, *CaMYB1*, *CaMYB2*, and *CaMYB3*, from *C. annuum* exhibited differential expression during fruit ripening (Guo et al., 2011).

*R2R3-MYB*s also play a specific regulatory role in the pepper capsaicin synthesis pathway. The *R2R3-MYB* transcription factor *CaMYB31* is therefore also a candidate to control pungency in *C. annuum*; it is known that Jasmonate-Inducible *CaMYB108*, a typical *R2R3-MYB* gene, regulates capsaicinoid biosynthesis and stamen development in *Capsicum* (Sun et al., 2019). Capsaicin *R2R3-MYB* candidate genes were screened in this study and analyzed using pepper RNA-seq data. This enabled investigation of *CaR2R3-MYB* DEGs in pericarp, placenta and seed, and placenta. The expression levels of the 26 up-regulated DEGs in the early stages of pericarp, placenta and seed, as well as placenta development are typically increased. This illustrates that up-regulated *CaR2R3-MYB* DEGs respond during capsaicin synthesis.

The expression of *CaR2R3-MYB* DEGs is tissue-specific during plant development. Partial *CaR2R3-MYB* DEGs have the highest expression levels in the pericarp, while other genes are expressed in the placenta, remarkable tissue-specific expression differences. Partial genes such as *Capana03g001205*, *Capana04g001328*, *Capana02g003369*, *Capana11g000757*, and *Capana09g002354* have almost detectable expressions in the early stages of both pericarp and placenta. Family genes only contain the duplicate gene pairs of *Capana02g000906-Capana02g003369* and *Capana02g000906-Capana06g001024*; in *Capsicum* species, several key CBGs are also expressed at early stages in the pericarp, placenta and seed, and placenta. In both phenylpropanoid and branched chain fatty acid pathways, expression levels of CBGs in this treatment are similar to those previously determined (Kim et al., 2014). Numerous *CaR2R3-MYB* family genes are related to CBGs in expression level, indicating that *CaR2R3-MYB* genes may directly or indirectly participate in the capsaicinoid biosynthesis.

In the co-expression network of capsaicinoid biosynthetic pathway, *MYB31* was co-expressed with 174 genes; amongst these co-expressed genes, 15 have been previously reported to be involved in CAPD biosynthesis (Zhu et al., 2019). Genes in the *CaR2R3-MYB* family co-expression network were identified; 19 were selected in the pericarp, placenta and seed, and placenta development process, which were co-expressed with CBGs that might be involved in capsaicinoid biosynthetic synthesis. Divergence expression of *CaR2R3-MYB* genes shape the pungent diversification in peppers. Six *CaR2R3-MYB* DEGs are candidate capsaicinoid biosynthetic related genes in this study, including *MYB31* and other five *CaR2R3-MYB*. This research systematically studied the main characteristics of the *CaR2R3-MYB* gene family, and also provides an important reference for the study of transcription factors related to the capsaicin synthesis pathway. This study provides comprehensive information about pepper *R2R3-MYB* genes and can help determine the function of pepper *R2R3-MYB* genes. It also provides important candidate genes for capsaicinoid biosynthesis research.

## Data Availability Statement

Publicly available datasets were analyzed in this study. This data can be found here: http://pepperhub.hzau.edu.cn/.

## Author Contributions

XZ, FL, XD, and JW designed the research. JW, YL, and BT conducted the experiments. JW and YL analyzed the data. JW wrote the manuscript. FL, LX, and JW revised the manuscript and improved the English. XZ and FL acquired the funding. All authors have read, reviewed, and approved the submitted version.

## Conflict of Interest

The authors declare that the research was conducted in the absence of any commercial or financial relationships that could be construed as a potential conflict of interest.

## Footnotes

1 Softberry: http://www.softberry.com

2 Gblocks: http://molevol.cmima.csic.es/castresana/Gblocks_server.html

3 “Zunla-1” Reference Genome: http://peppersequence.genomics.cn/

4 PlantCARE: http://bioinformatics.psb.ugent.be/webtools/plantcare/html/

5 WGCNA-R package: https://horvath.genetics.ucla.edu/html/Coexpression Network/Rpackages/WGCNA/index.html

6 GenScript: https://www.genscript.com/tools/real-time-pcr-taqman-primer-design-tool

## Supplementary Material

Supplementary Figure 1 The DEGs of *CaR2R3-MYB* in pepper between pericarp in different stages (adjust *P*-value < 0.01, | Log2foldchange| > 1). Orange bars are up-regulated genes and blue are down-regulated DEGs.

Supplementary Figure 2 qRT-PCR and RNA-seq data of four CBGs.

Supplementary Figure 3 Expression patterns of capsaicinoid-biosynthetic related *CaR2R3-MYB* genes.

Supplementary Table 1 The basic information of *R2R3-MYB* gene family in *Capsicum*. List of predicted genes and related information include gene name (Zunla_1), CM334 homologous gene and gene locus, molecular details, and predicting subcellular localization.

Supplementary Table 2 Genes and primers used in the quantitative real-time PCR.

Supplementary Table 3 *Cis*-regulatory elements annotations.
