# Corrigendum: Genome-wide identification and capsaicinoid biosynthesis-related expression analysis of the *R2R3-MYB* gene family in *Capsicum annuum* L.

**DOI:** 10.3389/fgene.2023.1240234

**Published:** 2023-06-29

**Authors:** Jin Wang, Yi Liu, Bingqian Tang, Xiongze Dai, Lingling Xie, Feng Liu, Xuexiao Zou

**Affiliations:** ^1^ College of Horticulture, Nanjing Agricultural University, Nanjing, China; ^2^ Engineering Research Center for Horticultural Crop Germplasm Creation and New Variety Breeding, Ministry of Education, Changsha, China; ^3^ Longping Branch, Graduate School of Hunan University, Changsha, China; ^4^ College of Horticulture, Hunan Agricultural University, Changsha, China; ^5^ Hunan Vegetable Research Institute, Changsha, China

**Keywords:** capsicum, *CaR2R3-MYB* family, capsaicinoid biosynthesis, expression analysis, co-expression

In the published article, there was an error regarding the **Affiliation** for Xuexiao Zou. As well as having affiliation 2 and 4, they should also have affiliation 1, College of Horticulture, Nanjing Agricultural University, Nanjing, China.

The authors apologize for this error and state that this does not change the scientific conclusions of the article in any way. The original article has been updated.

