# Supplementary figures and images for: Genome-Wide Identification and Capsaicinoid Biosynthesis-Related Expression Analysis of the R2R3-MYB Gene Family in Capsicum annuum L
Source: Front Genet. 2020 Dec 21;11:598183. doi: 10.3389/fgene.2020.598183 (PMC7779616; doi:10.3389/fgene.2020.598183)

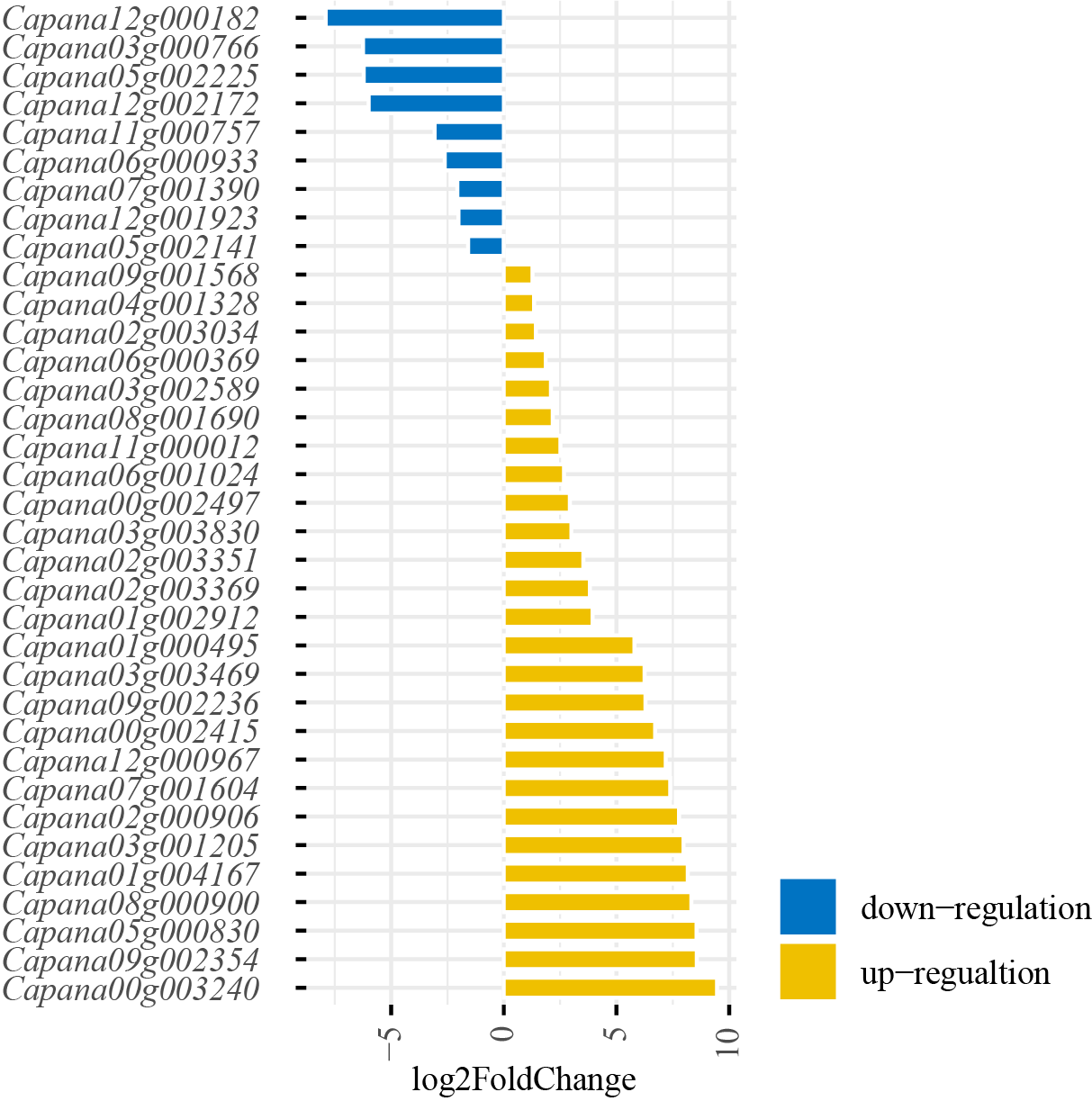

Supplement: Supplementary Figure 1 — The DEGs of CaR2R3-MYB in pepper between pericarp in different stages (adjust P-value < 0.01, | Log2foldchange| > 1). Orange bars are up-regulated genes and blue are down-regulated DEGs. [file Image_1.JPEG]

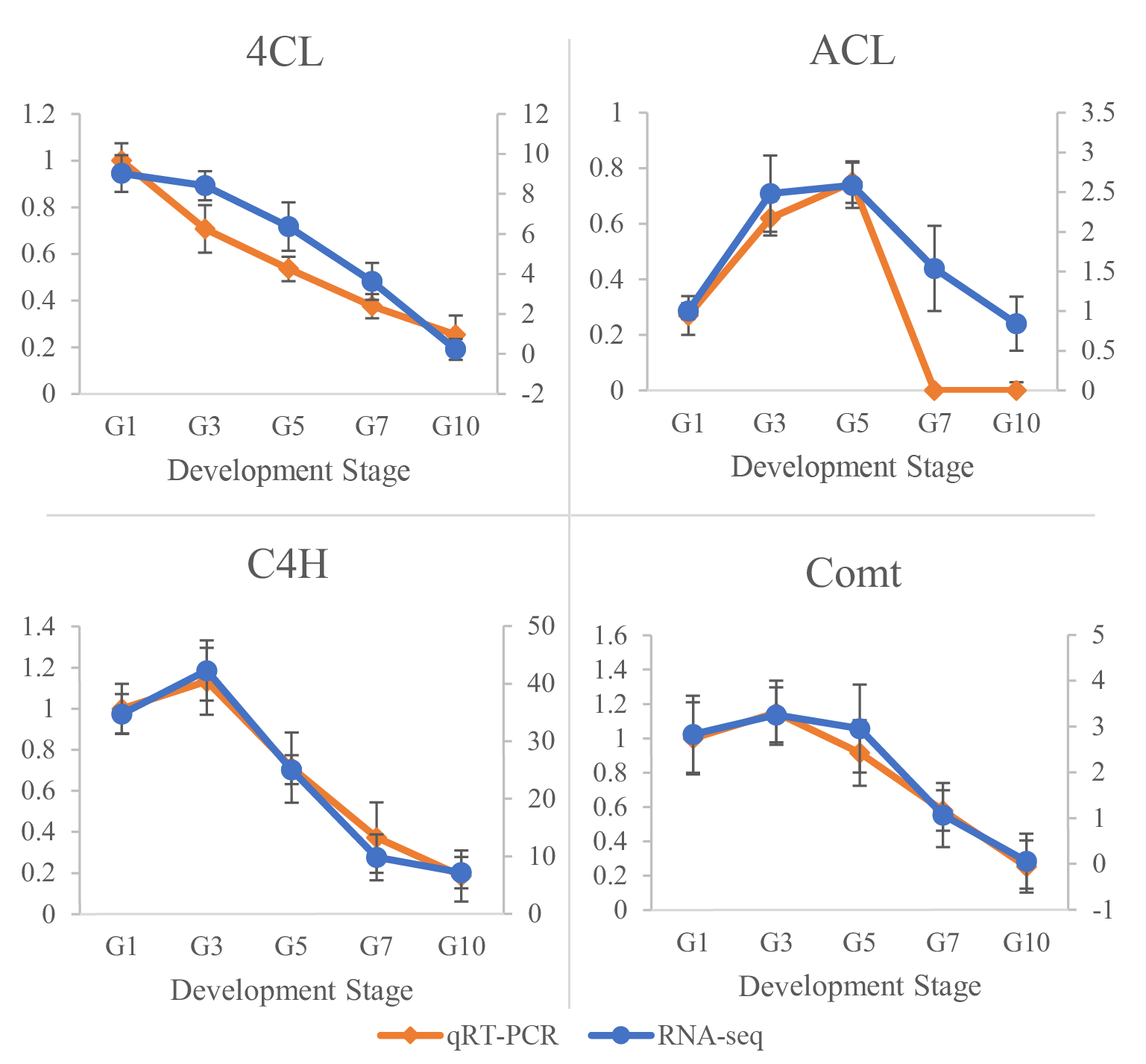

Supplement: Supplementary Figure 2 — qRT-PCR and RNA-seq data of four CBGs. [file Image_2.JPEG]

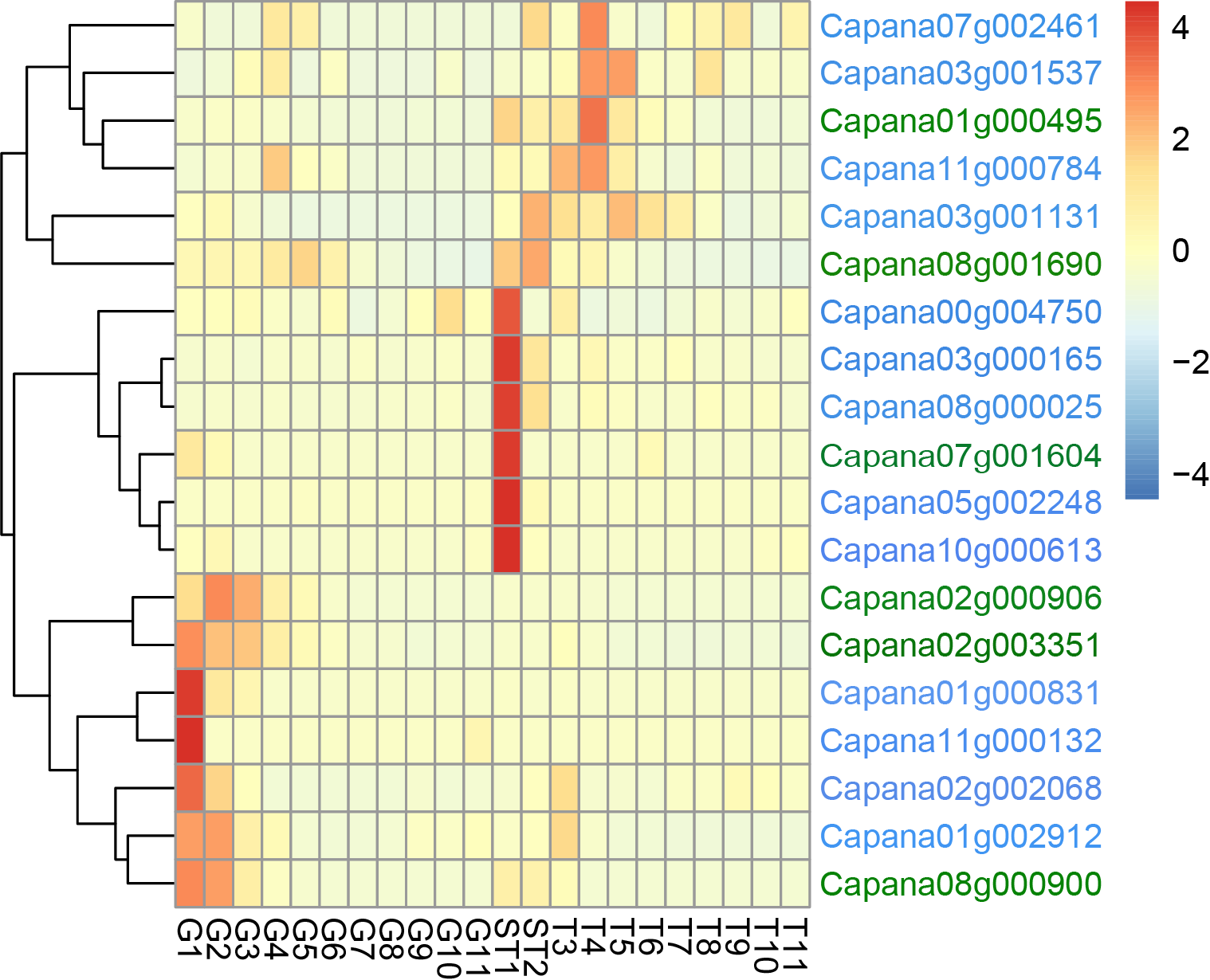

Supplement: Supplementary Figure 3 — Expression patterns of capsaicinoid-biosynthetic related CaR2R3-MYB genes. [file Image_3.JPEG]
