# Supplementary material for: Genome-Wide Identification and Capsaicinoid Biosynthesis-Related Expression Analysis of the R2R3-MYB Gene Family in Capsicum annuum L
Source: Front Genet. 2020 Dec 21;11:598183. doi: 10.3389/fgene.2020.598183 (PMC7779616; doi:10.3389/fgene.2020.598183)
Supplement: Supplementary Table 1 — The basic information of R2R3-MYB gene family in Capsicum. List of predicted genes and related information include gene name (Zunla_1), CM334 homologous gene and gene locus, molecular details, and predicting subcellular localization. [file Data_Sheet_1.PDF]

Table S1. The basic information of *R2R3-MYB* gene family in Capsicum. List of predicted genes and related information include gene name (Zunla_1), CM334 homologous gene and gene locus, molecular details, and predicting subcellular localization.

Gene Accession No.	CM334 homlog	Chr	Start	End	Strain	PI	Mw	Subcellular localization
Capana00g000426	*CA08g16690*	Chr00	245082979	245085150	-	9.78	33460.55	Nuclear
Capana00g000916	*CA11g10960*	Chr00	313181878	313186614	-	9.36	34548.68	Nuclear
Capana00g002415	*CA00g22820*	Chr00	475315945	475316785	-	8.54	23606.05	Nuclear
Capana00g002497	*CA00g59350*	Chr00	478813355	478814572	-	9.21	19921.69	Nuclear
Capana00g003154	*CA03g14200*	Chr00	529452992	529454126	+	9.36	27006.61	Nuclear
Capana00g003240	*CA07g03940*	Chr00	536861069	536861911	+	8.94	23684.17	Nuclear
Capana00g003294	*CA06g00130*	Chr00	539091578	539092530	-	6.05	27049.5	Nuclear
Capana00g003567	*CA06g10340*	Chr00	561136683	561141823	-	6.72	32273.23	Nuclear
Capana00g004750	*CA10g18410*	Chr00	667230015	667232200	+	6.7	39748.27	Nuclear
Capana00g004770	*CA10g18260*	Chr00	667984451	667985953	+	5.89	31259.75	Nuclear
Capana00g004862	*CA06g15120*	Chr00	671021059	671023014	-	8.94	31633.93	Nuclear
Capana01g000070	*CA01g00510*	Chr01	1058945	1060256	+	8.98	29811.89	Nuclear
Capana01g000184	*CA01g02130*	Chr01	2624079	2627319	+	5.87	38546.67	Nuclear
Capana01g000495	*CA01g04550*	Chr01	8532171	8534333	-	5.26	31703.49	Nuclear
Capana01g000831	*CA00g61570*	Chr01	18001252	18004368	+	4.87	36312.45	Cytoplasmic
Capana01g001063	*CA01g07370*	Chr01	29358044	29360280	-	5.18	34109.75	Nuclear
Capana01g001065	*CA01g07360*	Chr01	29440401	29441906	-	6.22	35118.3	Nuclear
Capana01g002201	*CA01g23890*	Chr01	127687353	127690271	-	9.22	37587.52	Extracellular
Capana01g002600	*CA01g20220*	Chr01	168571287	168574876	+	5.95	40758.17	Nuclear
Capana01g002912	*CA01g24400*	Chr01	184519881	184524874	-	5.13	45985.36	Nuclear
Capana01g004167	*CA01g32550*	Chr01	285916690	285918854	-	9.28	31520.62	Nuclear
Capana02g000670	*CA08g16690*	Chr02	82998297	83003405	-	8.37	31841.2	Cytoplasmic
Capana02g000906	*CA02g08640*	Chr02	101630051	101632638	+	5.44	36923.14	Nuclear
Capana02g000991	*CA02g00230*	Chr02	106006424	106010078	-	6.31	22561.17	Nuclear
Capana02g002068	*CA02g16960*	Chr02	139062508	139063948	+	5.77	43313.07	Nuclear
Capana02g002930	*CA02g24700*	Chr02	152836767	152838156	+	6.78	37441.75	Nuclear
Capana02g003034	*CA02g14840*	Chr02	154369720	154370743	-	7.09	32119.35	Nuclear
Capana02g003250	*CA02g27040*	Chr02	157972927	157974052	-	8.91	29489.39	Nuclear
Capana02g003351	*CA02g28060*	Chr02	159455402	159457441	-	5.65	45202.92	Nuclear
Capana02g003369	*CA02g28250*	Chr02	159795480	159796892	-	6.46	34330.36	Nuclear
Capana02g003511	*CA02g29740*	Chr02	161668218	161671033	-	7.06	23419.99	Nuclear
Capana02g003594	*CA02g30450*	Chr02	162617954	162619049	-	5.93	29406.03	Nuclear
Capana03g000165	*CA02g19560*	Chr03	2239290	2241210	-	7.24	42520.89	Nuclear

Capana03g000696	*CA03g33530*	Chr03	10280928	10282352	+	6.71	33707.52	Nuclear
Capana03g000766	*CA03g30090*	Chr03	11243966	11245291	-	6.25	36719.87	Nuclear
Capana03g001131	*CA03g27000*	Chr03	19303351	19312098	+	5.71	42881.79	Nuclear
Capana03g001205	*CA00g00420*	Chr03	20420520	20421902	+	5.55	37963.06	Nuclear
Capana03g001537	*CA03g23210*	Chr03	28772030	28775379	+	8.76	29316.58	Nuclear
Capana03g002269	*CA09g07680*	Chr03	56383549	56385481	-	9.01	20173.79	Nuclear
Capana03g002311	*CA07g03650*	Chr03	59886160	59888615	-	5.89	31764.22	Nuclear
Capana03g002589	*CA03g12790*	Chr03	87492084	87493272	+	7.71	33459.39	Nuclear
Capana03g002680	*CA11g14620*	Chr03	99504399	99505444	+	9.41	28829.98	Nuclear
Capana03g003469	*CA09g16760*	Chr03	223450334	223451768	-	8.26	25465.55	Nuclear
Capana03g003830	*CA00g88900*	Chr03	238881523	238883594	+	5.18	31499.29	Nuclear
Capana04g000607	*CA00g75700*	Chr04	9699385	9700365	+	9.31	35521.57	Nuclear
Capana04g000748	*CA04g16680*	Chr04	13537982	13539510	-	8.41	37347.24	Nuclear
Capana04g001058	*CA04g09100*	Chr04	28087234	28088702	-	5.63	39267.36	Nuclear
Capana04g001328	*CA04g13540*	Chr04	45054441	45057340	-	6.41	73888.44	Nuclear
Capana04g001901	*CA04g08580*	Chr04	133051924	133054288	-	6.63	37570.39	Nuclear
Capana04g002141	*CA11g04240*	Chr04	176594924	176597140	+	7.15	49674.97	Nuclear
Capana05g000213	*CA05g01900*	Chr05	3691912	3694396	+	6.39	36371.96	Nuclear
Capana05g000830	*CA12g02490*	Chr05	32014486	32016497	-	5.05	34511.62	Nuclear
Capana05g001776	*CA05g13580*	Chr05	174661990	174664465	+	6.27	40034.01	Nuclear
Capana05g002019	*CA05g15200*	Chr05	193370317	193371415	-	8.86	27970.67	Nuclear
Capana05g002141	*CA05g16020*	Chr05	200386436	200395273	-	5.15	44045.01	Nuclear
Capana05g002225	*CA05g16530*	Chr05	204661073	204663581	-	6.31	36803.74	Nuclear
Capana05g002248	*CA05g16780*	Chr05	205161625	205162757	+	10.18	16376.79	Nuclear
Capana05g002430	*CA02g16960*	Chr05	214996142	214998098	-	6.38	27031.08	Nuclear
Capana06g000131	*CA06g27890*	Chr06	1776563	1778125	+	4.76	32579.8	Nuclear
Capana06g000369	*CA06g25110*	Chr06	5294908	5296352	-	9.25	34505.55	Nuclear
Capana06g000460	*CA06g24340*	Chr06	6676996	6678375	+	6	26352.39	Nuclear
Capana06g000521	*CA00g82220*	Chr06	7763661	7766330	+	7.55	34256	Nuclear
Capana06g000933	*CA06g19980*	Chr06	16474649	16476299	-	6.37	36977.48	Nuclear
Capana06g000945	*CA09g07480*	Chr06	16644632	16647152	-	6.54	41711.73	Nuclear
Capana06g001024	*CA06g18990*	Chr06	17776705	17778561	+	5.73	41151.71	Nuclear
Capana06g002787	*CA06g03930*	Chr06	204409827	204413683	-	7.73	24265.29	Nuclear
Capana07g000044	*CA07g00390*	Chr07	1710607	1713167	-	5.91	38726.58	Nuclear
Capana07g000320	*CA02g02750*	Chr07	16120847	16133280	+	6.56	31859.3	Nuclear
Capana07g000392	*CA07g04070*	Chr07	25204182	25204648	+	8.93	12614.8	Nuclear
Capana07g001390	*CA00g64840*	Chr07	178238951	178241451	+	6.39	53825.74	Nuclear

Capana07g001603	*CA11g14610*	Chr07	192504459	192506350	+	6.25	28384.4	Nuclear
Capana07g001604	*CA11g14620*	Chr07	192576385	192577754	+	8.48	29642.94	Nuclear
Capana07g001609	*CA07g12950*	Chr07	192864497	192865869	-	5.99	26549.87	Nuclear
Capana07g001614	*CA07g12980*	Chr07	193028314	193034955	+	9.17	33643.61	Nuclear
Capana07g001626	*CA07g13030*	Chr07	194321446	194322672	-	5.84	38470.08	Nuclear
Capana07g001718	*CA07g14180*	Chr07	198298368	198300409	-	9.48	38633.25	Mitochondrial
Capana07g002461	*CA07g21180*	Chr07	220946392	220947906	-	6.6	33218.15	Nuclear
Capana08g000025	*CA08g00310*	Chr08	441291	443513	+	7.57	32946.82	Nuclear
Capana08g000900	*CA08g06950*	Chr08	121554202	121556633	-	6.52	34654.73	Nuclear
Capana08g001690	*CA00g53680*	Chr08	134763056	134764584	+	7.25	36997.04	Nuclear
Capana08g002728	*CA08g19210*	Chr08	151476418	151477753	-	9.01	31266.03	Nuclear
Capana09g000204	*CA09g16760*	Chr09	6689483	6692426	+	5.84	30466.7	Nuclear
Capana09g000548	*CA00g89710*	Chr09	20884747	20888450	+	5.87	53590.86	Nuclear
Capana09g001568	*CA09g06510*	Chr09	182613736	182615343	-	7.13	32657.37	Nuclear
Capana09g001653	*CA09g06330*	Chr09	191512140	191519434	+	5.24	38387.24	Cytoplasmic
Capana09g002236	*CA09g02000*	Chr09	234926605	234929253	-	6.31	34982.92	Nuclear
Capana09g002253	*CA09g01840*	Chr09	235225671	235226472	-	9.75	15010.4	Nuclear
Capana09g002354	*CA09g01270*	Chr09	237088354	237092075	+	6.67	55872.16	Nuclear
Capana09g002253	*CA10g01010*	Chr09	235225671	235226472	-	9.75	15010.4	Nuclear
Capana09g002354	*CA10g03650*	Chr09	237088354	237092075	+	6.67	55872.16	Nuclear
Capana10g000198	*CA10g05260*	Chr10	3547776	3551333	+	8.32	35616.72	Nuclear
Capana10g000447	*CA10g05760*	Chr10	14194173	14196268	+	6.76	37844.17	Nuclear
Capana10g000602	*CA10g11650*	Chr10	29047351	29049010	+	5.75	40929.5	Nuclear
Capana10g000613	*CA10g15640*	Chr10	30356499	30358217	-	7.58	36486.81	Nuclear
Capana10g001433	*CA11g20320*	Chr10	155518613	155520091	+	9.07	30231.43	Nuclear
Capana10g001872	*CA11g17990*	Chr10	189603391	189609160	-	5.8	42095.42	Mitochondrial
Capana11g000012	*CA11g13580*	Chr11	165807	174223	+	5.01	109766.43	Nuclear
Capana11g000132	*CA00g69840*	Chr11	3618160	3620157	-	8.68	35772.2	Nuclear
Capana11g000757	*CA11g11150*	Chr11	36160246	36161637	+	6.87	26233.44	Nuclear
Capana11g000784	*CA11g04380*	Chr11	39797237	39801454	-	6.06	40002.32	Nuclear
Capana11g001008	*CA11g04390*	Chr11	88859456	88860702	+	6.45	26000.52	Nuclear
Capana11g001805	*CA11g00850*	Chr11	196775482	196778878	+	8.8	39072.14	Nuclear
Capana11g001806	*CA12g21100*	Chr11	196801410	196803070	-	8.66	39422.6	Nuclear
Capana11g002314	*CA10g18260*	Chr11	218600081	218601555	+	5.71	33799.33	Nuclear
Capana12g000182	*CA00g61780*	Chr12	3013299	3014987	-	6.55	34184.58	Nuclear
Capana12g000748	*CA12g14080*	Chr12	21240187	21240915	+	8.27	27223.71	Nuclear
Capana12g000967	*CA12g08700*	Chr12	37891276	37893142	+	6.17	39602.05	Nuclear

Capana12g001140	*CA12g07260*	Chr12	48700880	48703608	-	6.47	73775.62	Nuclear
Capana12g001923	*CA08g16690*	Chr12	181340162	181342129	-	8.49	57403.54	Nuclear
Capana12g002172	*CA11g10960*	Chr12	202672223	202674447	+	5.88	25475.33	Mitochondrial
