# Supplementary material for: Genome-Wide Identification and Capsaicinoid Biosynthesis-Related Expression Analysis of the R2R3-MYB Gene Family in Capsicum annuum L
Source: Front Genet. 2020 Dec 21;11:598183. doi: 10.3389/fgene.2020.598183 (PMC7779616; doi:10.3389/fgene.2020.598183)
Supplement: Supplementary Table 2 — Genes and primers used in the quantitative real-time PCR. [file Data_Sheet_2.PDF]

Table S2. Genes and primers used in the quantitative real-time PCR

Gene name	Forward primer sequence 5'-3'	Reserve primer sequence 5'-3'	Product length(bp)
4CL	GGACCGATTGAAGGAATTGA	GGACAACAGCAGCATCAGAA	107
Acl	TTCCTTCAAGCACAACCAGA	GCGAGTAGCTGGCTTCATTC	94
C4H	CTTGGTTAACGCTTGGTGGT	CCGAATGGAAGGAATCTGAA	135
Comt	CCTGCGAATGGAAAAGTGAT	TCTTTGCCTCCTGGGTTATG	125
Capana08g001690	GCAGCTAGATTGCCTGGAAG	GACGCTTTCTCTTCGCTCTC	140
Capana02g003351	TGCACTGTTGTCCAATGACG	TGGACCTGAGCTTCGATTGT	122
Capana01g000495	GGAAGGCAACCTTGTTGTGA	CGAAGACCGGCAAGTTTAGG	137
Capana08g000900	GAGATCGGTGTTGCTGGTTC	ATGGCATTGGAGCTACAGGT	125
Capana02g000906	CCTCACGGACAGGCTTGATA	GCTACCCGAATTCACTGTCG	112
Capana07g001604	CATCACTTGAGTTTGTCGCATA	CTTGCCAGGAAGATCAGACA	95
Actin	GCCTAGAAATTTGAGCCTCATT	AAGATGGTATTGTTGCTGATGG	124
