# Supplementary material for: Genome-Wide Identification and Capsaicinoid Biosynthesis-Related Expression Analysis of the R2R3-MYB Gene Family in Capsicum annuum L
Source: Front Genet. 2020 Dec 21;11:598183. doi: 10.3389/fgene.2020.598183 (PMC7779616; doi:10.3389/fgene.2020.598183)
Supplement: Supplementary Table 3 — Cis-regulatory elements annotations. [file Data_Sheet_3.PDF]

Table S3. Cis-regulatory elements annotations.

Cis-elements	Annotation	Annotation Functions
CAT-box	Cellular Development	cis-acting regulatory element related to meristem expression
CCAAT-box	Cellular Development	MYBHv1 binding site
GCN4_motif	Cellular Development	cis-regulatory element involved in endosperm expression
HD-Zip 1	Cellular Development	element involved in differentiation of the palisade mesophyll cells
MBSI	Cellular Development	MYB binding site involved in flavonoid biosynthetic genes regulation
MSA-like	Cellular Development	cis-acting element involved in cell cycle regulation
NON-box	Cellular Development	cis-acting regulatory element related to meristem specific activation
RY-element	Cellular Development	cis-acting regulatory element involved in seed-specific regulation
A-box	Hormone	sequence conserved in alpha-amylase promoters
ABRE	Hormone	cis-acting element involved in the abscisic acid responsiveness responsiveness
AuxRE	Hormone	part of an auxin-responsive element
AuxRR-core	Hormone	cis-acting regulatory element involved in auxin responsiveness
CGTCA-motif	Hormone	cis-acting regulatory element involved in the MeJA-responsiveness
GARE-motif	Hormone	gibberellin-responsive element
O2-site	Hormone	cis-acting regulatory element involved in zein metabolism regulation
P-box	Hormone	gibberellin-responsive element
SARE	Hormone	cis-acting element involved in salicylic acid responsiveness
TATC-box	Hormone	cis-acting element involved in gibberellin-responsiveness
TCA-element	Hormone	cis-acting element involved in salicylic acid responsiveness
TGACG-motif	Hormone	cis-acting regulatory element involved in the MeJA-responsiveness
TGA-element	Hormone	auxin-responsive element
3-AF1 binding site	Stress	light responsive element
AAAC-motif	Stress	light responsive element
ACE	Stress	cis-acting element involved in light responsiveness
ARE	Stress	cis-acting regulatory element essential for the anaerobic induction induction
ATC-motif	Stress	part of a conserved DNA module involved in light responsiveness
ATCT-motif	Stress	part of a conserved DNA module involved in light responsiveness
AT-rich element	Stress	element for maximal elicitor-mediated activation (2copies)
Box 4	Stress	part of a conserved DNA module involved in light responsiveness
circadian	Stress	cis-acting regulatory element involved in circadian control
G-Box	Stress	cis-acting regulatory element involved in light responsiveness
GC-motif	Stress	enhancer-like element involved in anoxic specific inducibility
GT1-motif	Stress	light responsive element
LTR	Stress	cis-acting element involved in low-temperature responsiveness
MBS	Stress	MYB binding site involved in drought-inducibility
MRE	Stress	MYB binding site involved in light responsiveness

Sp1	Stress	light responsive element
TC-rich repeats	Stress	cis-acting element involved in defense and stress responsiveness
WUN-motif	Stress	wound-responsive element
